# A comparison of pointing movement kinematics between virtual and physical environments

**DOI:** 10.1007/s00221-025-07162-0

**Published:** 2025-10-09

**Authors:** Shinji Yamamoto, Gavin Buckingham, Tom Arthur, David Harris

**Affiliations:** 1 Graduate School of Sport Sciences, Nihon Fukushi University, Chita-Gun, Japan; 2 School of Public Health and Sport Sciences, University of Exeter, Exeter, UK

**Keywords:** Virtual reality, VR, Real world, Gravity, Motor control, Reaching

## Abstract

Humans control their body movements by exploiting gravity to minimise muscle effort while achieving task goals. Most of these findings have been observed in physical environments, although some have also been confirmed in virtual environments. However, research using virtual environments to explore gravity-related motor control mechanisms has yet to directly compare motor performance between virtual and physical environments. Therefore, the present study aimed to examine in detail the potential differences in upper-limb pointing movements between virtual and physical environments. To this end, participants performed pointing tasks in four directions (upward, downward, leftward, and rightward, from an allocentric perspective) in both upright and lying postures, under both virtual and physical conditions. Our results showed that relative duration to peak velocity—a well-established kinematic indicator of gravity utilisation—was consistently shorter for upward than for downward movements across both environments and both postures. However, no differences were observed between the two environments when posture and movement direction were held constant. Furthermore, no differences were observed between the environments in terms of whole velocity and acceleration profiles, as well as in movement duration, peak velocity, peak acceleration, peak deceleration, and the relative durations to peak acceleration and peak deceleration. The similarity in relative duration to peak velocity between virtual and physical environments suggests that the effects of gravity on pointing movements can be reliably assessed in virtual environments as in physical ones. This supports the use of virtual environments as valid tools for studying pointing movements.

## Introduction

A substantial body of research has explored how humans control their movements under the influence of gravity (for review, Jörges and López-Moliner 2017; White et al. 2020). For example, studies employing upper-limb pointing tasks have revealed that humans consider and take advantage of the gravitational effect to minimise muscle effort (Gaveau et al. 2016, 2021; Papaxanthis et al. 2003; Poirier et al. 2022). A key kinematic indicator of gravity utilisation is the shape of the velocity curve, particularly the relative duration to peak velocity (rDPV). It has been consistently reported that the rDPV is shorter for upward movements compared to downward movements, while no differences are typically observed between leftward and rightward movements (Gaveau et al. 2014, 2021; Gentili et al. 2007; Papaxanthis et al. 2005; Yamamoto and Kushiro 2014). In addition, these direction-dependent differences gradually diminish when humans are exposed to microgravity (Papaxanthis et al. 2005; Gaveau et al. 2016). These findings indicate that humans adopt movement strategies that exploit gravity: specifically, by prolonging the deceleration phase during upward movements (to brake), and the acceleration phase during downward movements (to enhance acceleration), they minimise muscle effort while achieving task goals.

Most research, including the studies mentioned above, has been conducted in physical environments to investigate how humans account for gravitational effects on upper-limb movements. However, some studies have utilised virtual environments, which offer the advantage of enabling experimental manipulation of environmental conditions that would be difficult or impossible to achieve in physical settings. For instance, Sciutti et al. (2012) used a virtual environment in which the motion of the fingertip during pointing was displayed as visual feedback on a screen. When the direction of the physical finger movement matched that of the visual feedback, rDPV was shorter for upward than for downward movements, with no differences observed between leftward and rightward directions. Furthermore, when the directions of the physical movement and the visual feedback were mismatched (e.g., an upward physical movement was visually presented as downward), the kinematics of the pointing movement were biased toward the visual feedback direction. Thus, virtual environments can serve as a powerful tool for advancing our understanding of how humans account for gravity in motor control. However, concerns have been expressed in the literature about whether perception and action in virtual tasks are fully representative of the physical equivalent (Harris et al. 2019). Virtual environments present a number of unusual challenges for the perceptual system, such as artificial presentation of egocentric distance cues and missing, uncertain, or unreliable haptics (Wann et al. 1995; Giesel et al. 2020). Several previous studies have reported that movements in a virtual environment tend to be slower and more exaggerated compared to that in a physical environment (Arlati et al. 2022; Furmanek et al. 2019; Magdalon et al. 2011). For example, Furmanek et al. (2019) examined kinematics of prehension movements and found that the movements in a virtual environment were slower than those in a physical environment, suggesting that differences in motor behaviour exist between the two visual environments.

Consequently, while virtual environments offer an exciting opportunity to study human behaviour in new ways (Buckingham 2019; Snow and Culham 2021), there is a real possibility that motor performance in virtual environments is fundamentally different to the physical environments. This would present a significant problem for the previous studies using virtual environments for upper-limb movement, as well as wider applications of virtual environments in psychological experimentation and applied interventions. For example, differences in gravity-considering motor control strategies between virtual and physical environments may pose problems when using a virtual environment for patient rehabilitation services, as strategies trained in a virtual environment may be difficult to apply or transfer to those required in a physical environment. Nevertheless, research using virtual environments to explore gravity-related motor control mechanisms has yet to directly *compare* motor performance between a virtual environment and a physical environment. Therefore, the present study aimed to examine in detail the potential differences in upper-limb pointing movements between virtual and physical environments. To this end, we had participants perform pointing tasks toward four directions (upward, downward, leftward, and rightward in allocentric perspective) in both upright and lying postures, under both virtual and physical environment conditions. We systematically assessed the differences in motor performance by adopting the approach of Le Seac’h and McIntyre (2007) and Gaveau et al. (2021), setting four movement directions and examining whether gravity-exploiting movement features emerged in both upright and lying postural conditions. For example, Le Seac’h and McIntyre (2007) had participants perform pointing movements in four directions under upright and lying postures, and showed that the rDPV was shorter for upward than downward movements regardless of posture, with no differences between leftward and rightward directions. Based on this, we considered that incorporating these four movement directions and two postures into this study protocol would allow for a systematic investigation of the kinematic differences in pointing movements between virtual and physical environments. By directly comparing gravity-related motor behaviour across virtual and physical environments, this study provides the first systematic test of whether well-established kinematic signatures of gravity exploitation are preserved in virtual environments. Furthermore, we computed additional kinematic parameters (e.g., relative duration to peak acceleration, relative duration to peak deceleration; see Sect. “Data Analysis”) to more thoroughly investigate potential differences in pointing kinematics between physical and virtual environments. In particular, to examine possible differences across the entire time course of movement, we conducted an exploratory analysis using Statistical Parametric Mapping (SPM). This study represents an essential step for validating virtual and augmented reality technologies as reliable tools for studying motor control and informing future applications in training, rehabilitation, and neuroscience.

Our hypotheses were preregistered prior to data analysis and can be found here: 10.17605/OSF.IO/EDVCT. Although prior findings suggest that movements in virtual environments can be slower and more exaggerated than in physical environments (Arlati et al. 2022; Brock et al. 2023; Furmanek et al. 2019), at the time of preregistration this evidence was not considered sufficiently strong for directional predictions about our primary dependent variable (H1), so we opted for a non-directional hypothesis. Despite anticipating some differences in movement kinematics between virtual and physical environments, we still expected gravity induced direction-dependent differences in rDPV between upward and downward movements (H2), but no differences between rightward and leftward movements (H3), consistent with previous studies conducted in physical environments, and as suggested by Sciutti et al. (2012).

### Hypothesis 1 (H1)

There will be a difference in rDPV between virtual and physical environments for both upward/downward and leftward/rightward movements.

### Hypothesis 2 (H2)

There will be difference in rDPV between upward and downward movements, both in virtual and physical environments.

### Hypothesis 3 (H3)

There will be no difference in rDPV between leftward and rightward movements, both in virtual and physical environments.

## Methods

### Participants

Twenty-eight, right-handed adults (mean age = 20.25 years, SD =  ± 3.32 years, 9 females, 19 males) free from sensorimotor disorders, history of vertigo, and previous negative response to virtual environments were recruited from the staff and student population at the University of Exeter. The sample size was determined a priori using G*Power based on detecting a moderate effect size (*η*_*p*_^2^ = 0.06) with 90% power in a repeated measures ANOVA (α = 0.05). The chosen effect size represents a conservative estimate, as previous studies have reported large effect sizes for rDPV—a key kinematic parameter in this study (*η*_*p*_^2^ = 0.14 in Poirier et al. 2022; *η*_*p*_^2^ = 0.42 in the preprint by Poirier et al. 2023). We chose to power the study to be sensitive to substantially smaller differences, so as to rule out all but small divergences between the two environments. Therefore, we conducted the power analysis to detect an effect size that is considered moderate (*η*_*p*_^2^ = 0.06). Handedness was assessed using the Edinburgh Handedness Inventory (Oldfield 1971), with participants showing a mean laterality quotient of 78.55 ± 24.43. All participants provided written informed consent and received £25 as compensation for their participation. The study was conducted in agreement with the principles of the Declaration of Helsinki (2013) and was approved by local Ethics Review Board at the Department of Public Health and Sport Sciences, University of Exeter (#7871984).

### Apparatus and setup

**Fig. 1:**
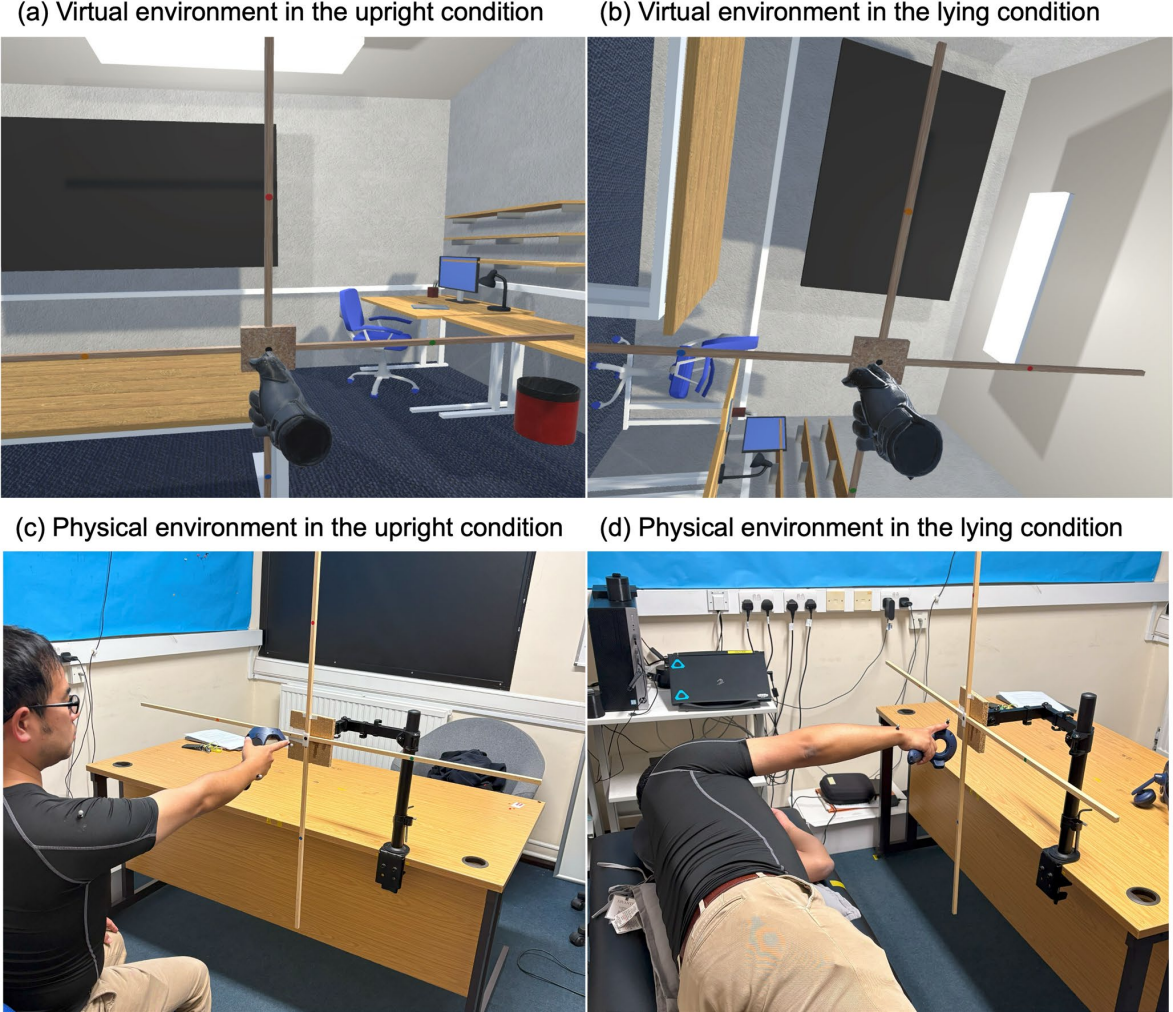
Experimental setup in the virtual (**a** and **b**) and physical environments (**c** and **d**), under two postural conditions: upright (**a** and **c**) and lying (**b** and **d**). In each condition, participants performed pointing movements from a start position (black dot indicated by a cross) toward one of four coloured targets (red, blue, green, and orange dots)

In the physical environment, an apparatus consisting of a cross-shaped structure indicating the start position and four target positions (upward, downward, leftward, and rightward in allocentric perspective) was set up (Le Seac’h and McIntyre 2007; Gaveau et al. 2021). The distance from the start position to each of the target positions was 30 cm. The virtual environment was a bespoke application developed using Unity (version 2019.2.12; Unity Technologies, San Francisco, CA) and replicated a room similar to the physical environment, featuring a cross with a start position and four targets (see Fig. 1). The distance between the start position and each target in the virtual environment was also set to 30 cm. To ensure spatial alignment between the physical and virtual environments, a tracker (HTC VIVE Tracker 3.0; HTC Inc., Taoyuan City, Taiwan) representing the center of the cross in the virtual environment was placed at the center of the cross in the physical environment. In the virtual environment, a head-mounted display (HMD; HTC VIVE Pro Eye) was used for displaying the virtual visual scene, and an HTC VIVE hand-held controller was used to represent the participant's hand in the virtual environment. Reflective markers were attached to the participants’ right upper limb (shoulder: acromion, elbow: lateral epicondyle, wrist: ulnar styloid process, and index finger: finger nail), and the movements of these markers during the pointing task were captured in three-dimensional space at 120 Hz with an 8-camera OptiTrack Flex 13 system (NaturalPoint, Inc., Corvallis, USA).

### Task and procedure

Each participant performed pointing movements toward four targets in both physical and virtual environments, under two postural conditions: upright and lying. In each combination of posture and environment, participants completed four blocks, with each block consisting of three trials per movement direction (i.e., three trials × four directions), resulting in a total of 16 blocks across the experiment. Short breaks were provided between blocks to prevent muscle fatigue due to repeated pointing movements. The order of posture and environmental conditions was counterbalanced across participants, and the sequence of movement directions within each block was randomised. Before starting the blocks in each posture and environmental condition, participants performed at least four practice trials (one trial for each direction). In each trial of each condition, participants were first informed of the target direction. They were then pointed at the start position (black dot) with their index finger, maintained that posture, and visually fixated on the designated target. Upon a verbal cue from the experimenter, participants performed the pointing movement toward the target and held the final arm posture until further instruction was given. They were instructed to execute the pointing movement with their arm extended (i.e., 1 degree of freedom: shoulder rotation), as quickly and accurately as possible, and to avoid making corrective movements during execution. To ensure a consistent load on the hand across all postural and visual environmental conditions, participants also held a controller in their hand throughout the experiment.

### Data analysis

The positional data obtained from the reflective markers were processed using a third-order low-pass Butterworth filter with a cutoff frequency of 5 Hz, implemented using the “butter” and “filtfilt” functions (Poirier et al. 2022). All kinematic parameters described below were derived from the marker attached to the index finger. Velocity and acceleration were calculated by applying a three-point numerical differentiation method to the index finger’s displacement data. The onset and offset of movement in each trial were defined as the first and last time points at which the index finger's velocity exceeded 10% of its peak value (Poirier et al. 2022).

To investigate how participants control their arm movements while taking care of the gravitational effects on motor control in virtual and physical environments, we first calculated two fundamental kinematic parameters: peak velocity (PV) and movement duration (MD; defined as the duration from movement onset to offset). We then computed the rDPV, defined as the duration from movement onset to peak velocity divided by MD and multiplied by 100. This index has been widely used in previous studies to index gravity utilisation, as mentioned in the Introduction section. To further examine the kinematics in greater detail, we analysed the acceleration profile to extract additional parameters: peak acceleration (PA), peak deceleration (PD), the relative duration to peak acceleration (rDPA; duration from movement onset to peak acceleration divided by MD and multiplied by 100), and the relative duration to peak deceleration (rDPD; time from movement onset to peak deceleration divided by MD and multiplied by 100). These normalised temporal parameters allow for direct comparison of the shapes of velocity and acceleration profiles across conditions, including virtual and physical environments.

For the statistical analysis of the kinematic parameters described above, we first assessed normality using the Shapiro–Wilk test in SPSS (IBM Corporation). If the assumption of normality was met, we conducted a three-way repeated-measures ANOVA with the following within-participant factors: visual environment (virtual vs. physical), movement direction (upward, downward, leftward, and rightward, defined in allocentric coordinates), and posture (upright vs. lying). When significant interactions among two or three factors were identified, we conducted simple main effects analysis and post hoc comparisons. If the assumption of normality was violated, we used the non-parametric Friedman test. When the Friedman test yielded significant results, we performed post hoc comparisons using the Wilcoxon signed-rank test. A significance level of 0.05 was adopted for all statistical tests, and Bonferroni correction was applied to adjust for post-hoc comparisons. When we performed post hoc comparisons, not all possible pairwise comparisons were tested. Specifically, comparisons between vertical (upward/downward) and lateral (leftward/rightward) directions, were not conducted, as they have not been commonly addressed in previous studies and were deemed unnecessary for the purpose of this study. Instead, we performed the following 16 planned comparisons. Accordingly, for these 16 comparisons, the significance threshold was adjusted to *p* = 0.003125 (0.05/16).

Pairs for comparisons between virtual and physical environments:

1. Upright-Virtual-Upward versus
  Upright-Physical-Upward
2. Upright-Virtual-Downward versus
  Upright-Physical-Downward
3. Upright-Virtual-Rightward versus
  Upright-Physical-Rightward
4. Upright-Virtual-Leftward versus
  Upright-Physical-Leftward
5. Lying-Virtual-Upward versus
  Lying-Physical-Upward
6. Lying-Virtual-Downward versus
  Lying-Physical-Downward
7. Lying-Virtual-Rightward versus
  Lying-Physical-Rightward
8. Lying-Virtual-Leftward versus
  Lying-Physical-Leftward

Pairs for comparisons between movement directions within each visual environment:

9. Upright-Virtual-Upward versus
  Upright-Virtual-Downward
10. Upright-Virtual-Rightward versus
  Upright-Virtual-Leftward
11. Upright-Physical-Upward versus
  Upright-Physical-Downward
12. Upright-Physical-Rightward versus
  Upright-Physical-Leftward
13. Lying-Virtual-Upward versus
  Lying-Virtual-Downward
14. Lying-Virtual-Rightward versus
  Lying-Virtual-Leftward
15. Lying-Physical-Upward versus
  Lying-Physical-Downward
16. Lying-Physical-Rightward versus
  Lying-Physical-Leftward

To extend the summary kinematic measures during pointing, we additionally used statistical parametric mapping (SPM) to conduct an exploratory, time-resolved analysis of the full movement trajectory, enabling the identification of subtle, temporally specific differences between virtual and physical environments. The kinematic parameters described above capture essential features of movement and serve as important kinematic landmarks for investigating differences between virtual and physical environments. However, these parameters are based on specific temporal points in the velocity and acceleration profiles—namely PA, PV, and PD. As such, they do not capture kinematic characteristics across other temporal intervals (e.g., which periods show differences in velocity before reaching peak velocity). To address this limitation, we additionally employed SPM analysis. SPM is a statistical technique originally developed in neuroimaging (Penny et al. 2011) and later adapted for biomechanical and motor control research (Brock et al. 2023; Pataky 2010; Robinson et al. 2015). It enables continuous comparisons of temporal series data, thereby allowing statistical inference over the entire kinematic profile rather than only at specific discrete temporal points. By applying SPM to the velocity and acceleration profiles, we aimed to explore more detailed temporal differences in movement between virtual and physical environments that may not be captured by traditional discrete kinematic measures. In this SPM analysis, the 16 comparisons mentioned above were conducted for both the velocity and acceleration profiles, with the statistical significance level set at 0.05.

## Results

Due to the loss of data from one participant, data from 27 participants were used for the analysis. Of the total 5184 trials conducted by these participants, three trials were excluded: two trials in which the MD exceeded the mean by more than three standard deviations (in the lying-virtual-leftward and lying-physical-rightward conditions, each from a different participant), and one trial with missing motion data (in the lying-physical-upward condition). The analysis was therefore conducted on the remaining 5181 trials. The means of the kinematic parameters across participants for each posture, environment, and movement direction are presented in Tables 1 and 2.

**Table 1:**
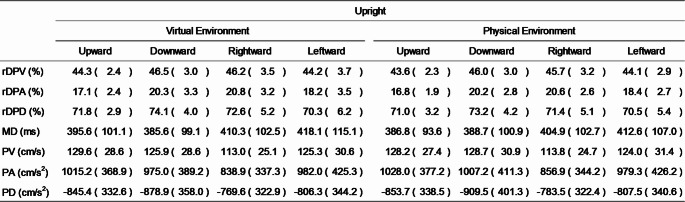
Means (standard deviations) of kinematic parameters in the upright condition

rDPV, relative duration to peak velocity; rDPA, relative duration to peak acceleration; rDPD, relative duration to peak deceleration; MD, movement duration; PV, peak velocity; PA, peak acceleration; PD, peak deceleration

**Table 2:**
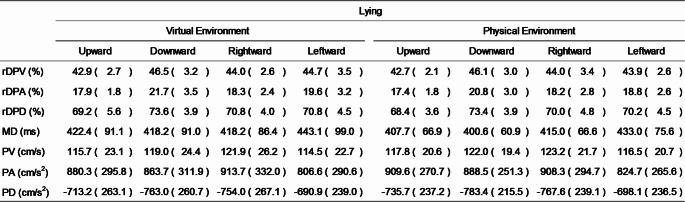
Means (standard deviations) of kinematic parameters in the lying condition

rDPV, relative duration to peak velocity; rDPA, relative duration to peak acceleration; rDPD, relative duration to peak deceleration; MD, movement duration; PV, peak velocity; PA, peak acceleration; PD, peak deceleration

**Fig. 2:**
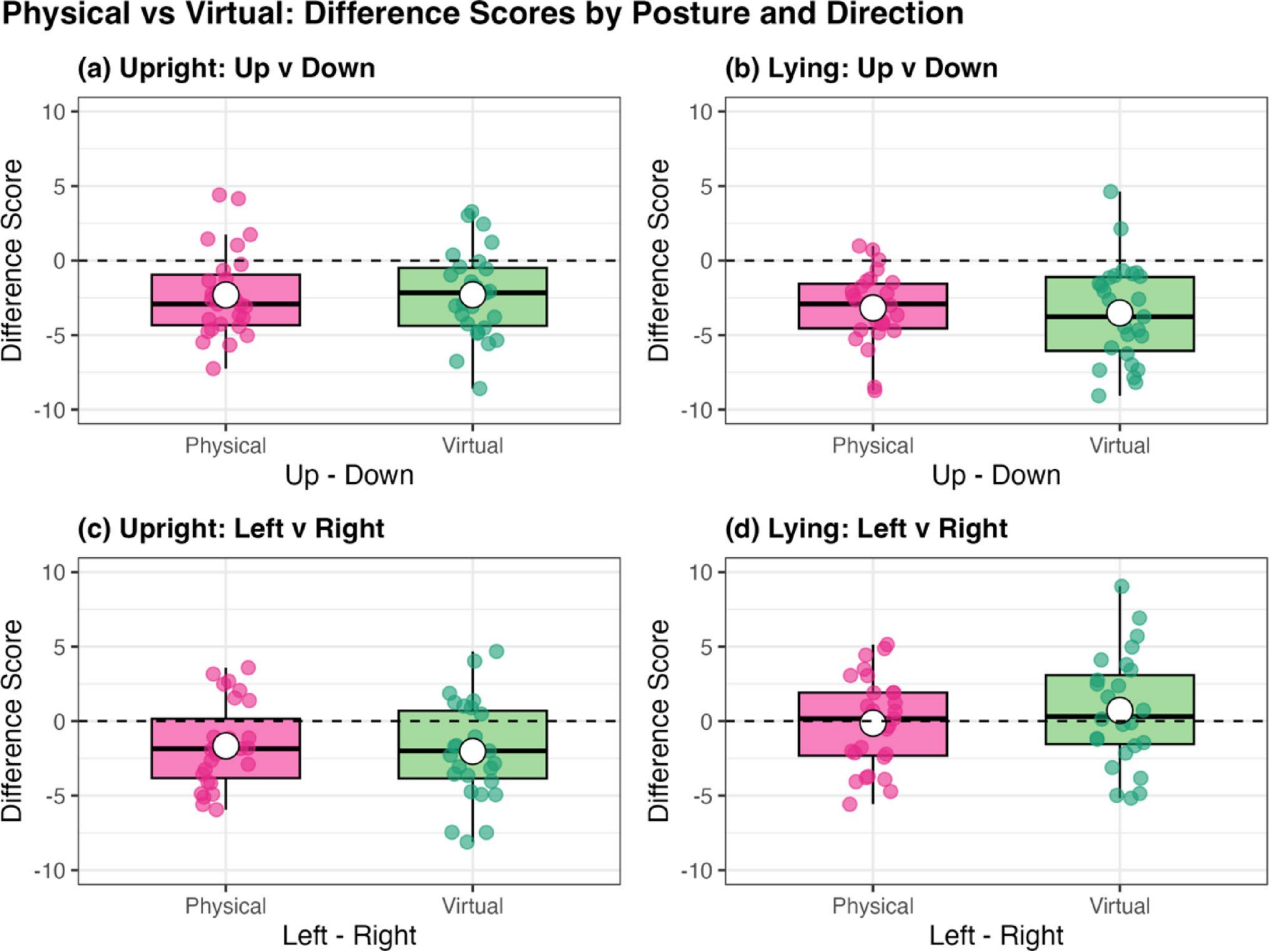
Difference scores in relative duration to peak velocity (rDPV) comparing physical and virtual environments across movement directions and postures. Each panel shows the difference score (up minus down or left minus right) as boxplots (median line, interquartile range box, whiskers extending 1.5 × IQR, individual points showing participants’ values, and white dot for the mean) for physical (pink) and virtual (green) environments

**Fig. 3:**
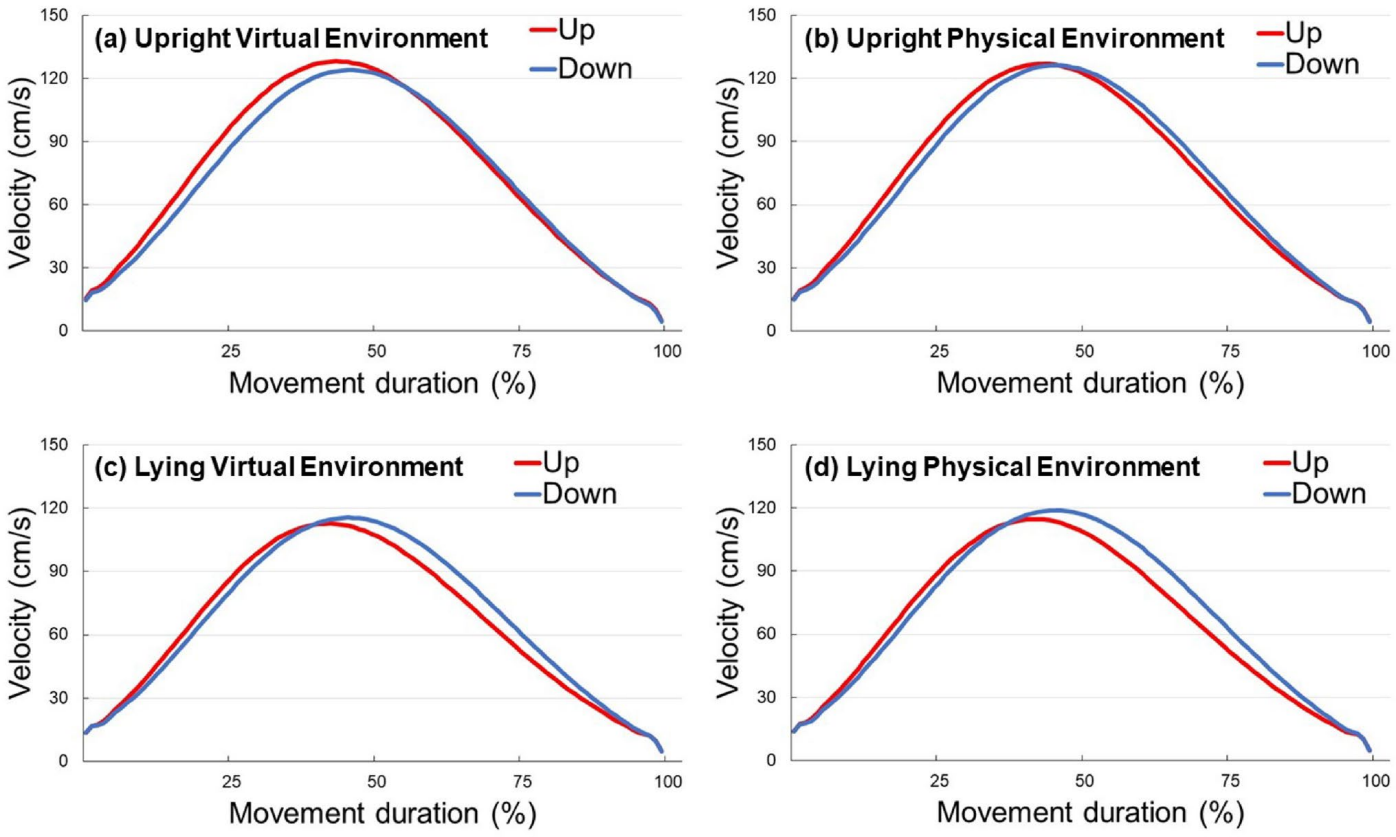
Mean velocity profiles for upward and downward movements in virtual and physical environments under upright and lying postures

**Fig. 4:**
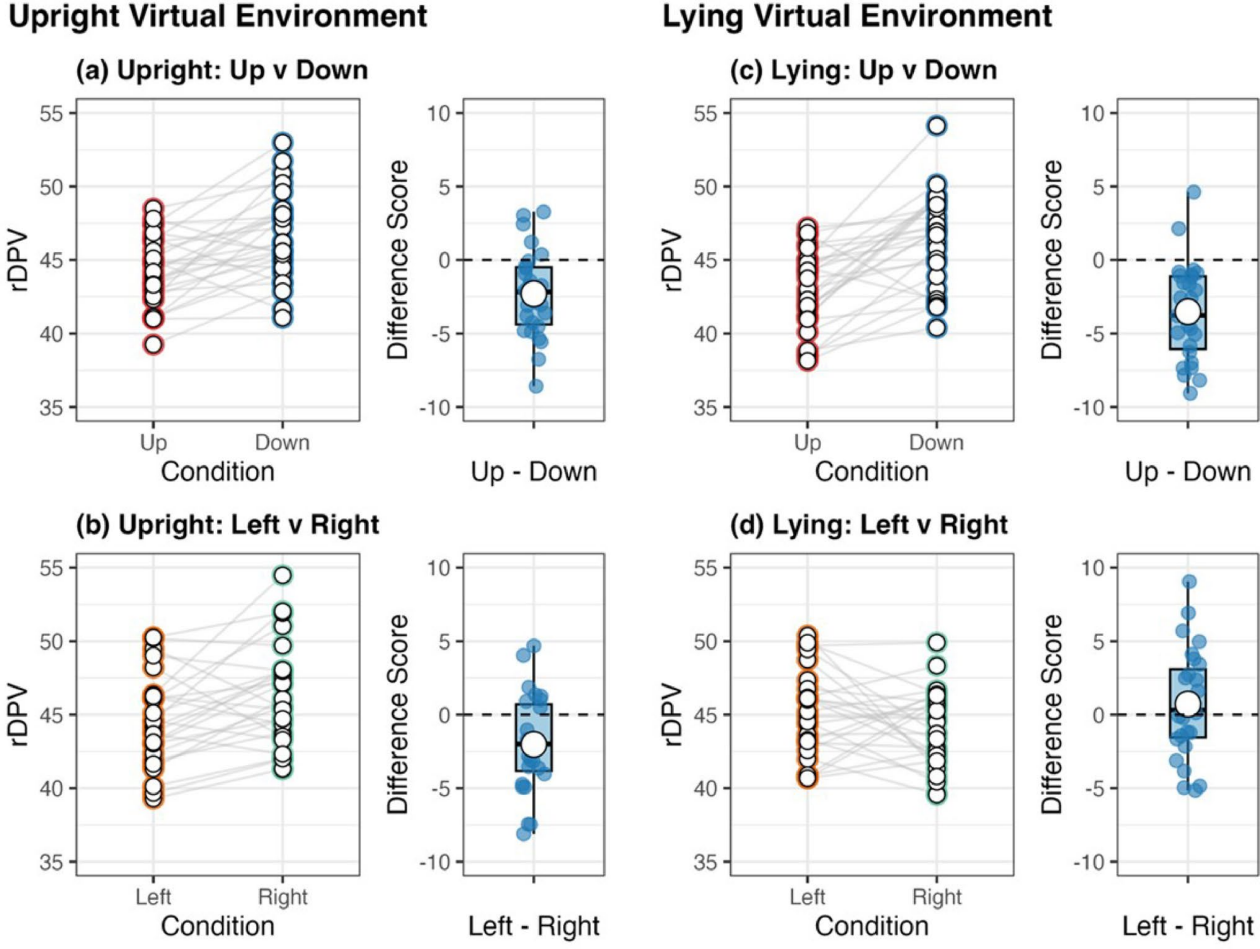
Comparison of rDPV for upward vs. downward and leftward vs. rightward for both postures in the virtual environment. In each of panels (**a**) to (**d**), the circles in the left panel represent individualparticipants’ data, with lines connecting the data for each movement direction within each participant. The right panel shows boxplot of the difference between movement directions, and the circle indicates the across-participant mean of the difference

**Fig. 5:**
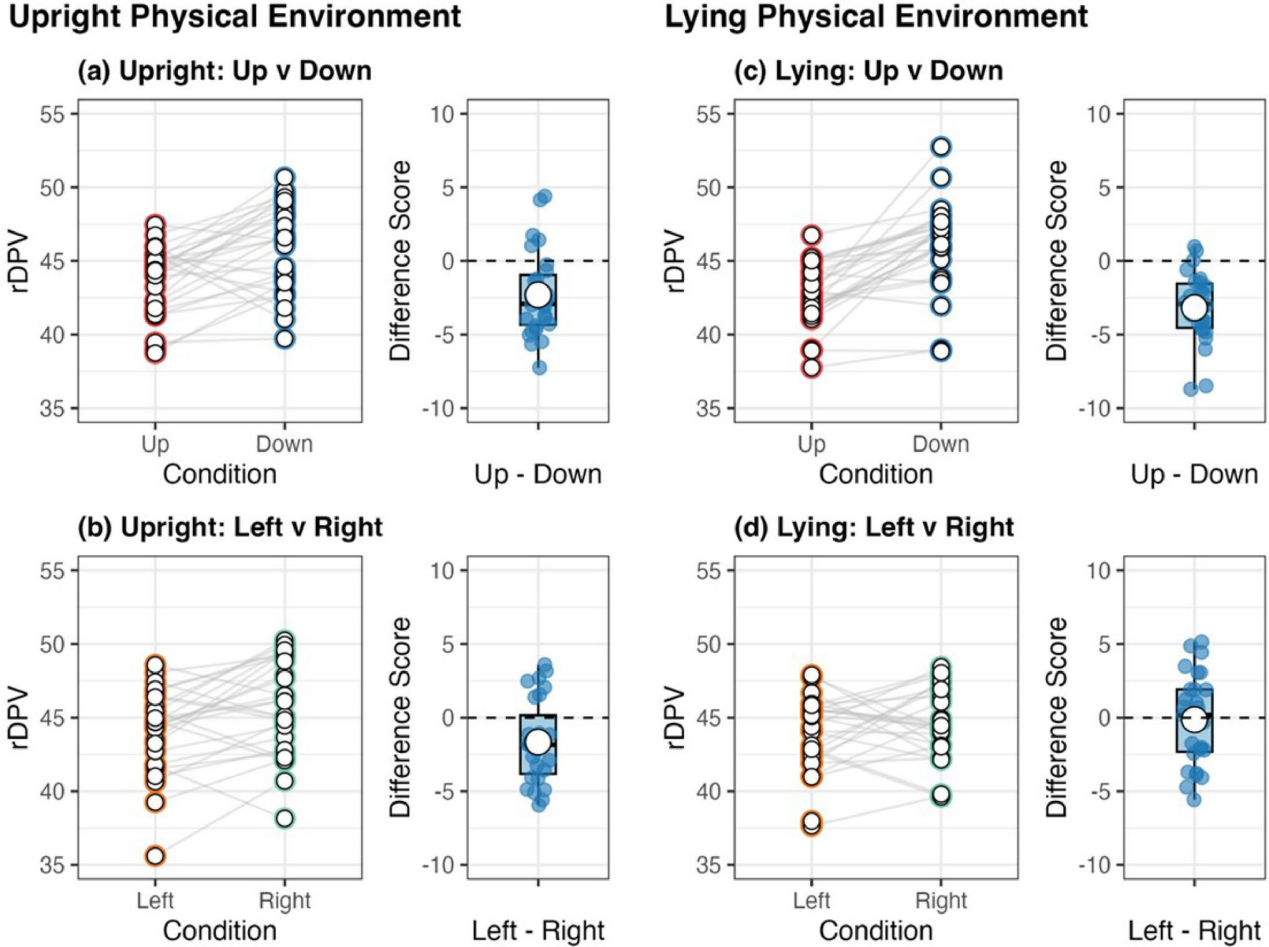
Comparison of rDPV for upward vs. downward and leftward vs. rightward for both postures in the physical environment. In each of panels (**a**) to (**d**), the circles in the left panel represent individual participants’ data, with lines connecting the data for each movement direction within each participant. The right panel shows boxplot of the difference between movement directions, and the circle indicates the across-participant mean of the difference

The normality assumption was not met for rDPV across all conditions, therefore, a Friedman test was conducted in this instance. Friedman’s test revealed significance in the data that required further exploration (*p* < 0.001), thus Wilcoxon signed-rank tests were subsequently used for pairwise comparisons between conditions (see Sect. “Data Analysis” for the list of comparison combinations). Comparisons between the virtual and physical environment conditions under the same posture and movement direction (Comparisons No. 1–8) revealed no significant differences (see Fig. 2).^1^ However, in both posture conditions, rDPV was significantly shorter for upward movements than for downward movements in *both* the virtual and physical environment conditions (all *p* < 0.002; see Figs. 3, 4, 5). No significant differences were observed between rightward and leftward movements across all posture and visual environmental conditions, but for the upright virtual environment condition, the result was close to the significant threshold of *p* = 0.003125 (*p* = 0.003126 for this condition; *p* = 0.012 for the upright physical environment condition; *p* = 0.400 for the lying virtual environment condition; *p* = 0.792 for the lying physical environment condition).

To assess the shapes of velocity and acceleration curves, a three-way repeated-measures ANOVA was conducted for rDPA (which met the assumption of normality). The results did not indicate any significant interactions across three factors (*p* = 0.485), between the visual environment and posture factors (*p* = 0.152), and between the visual environment and movement direction factors (*p* = 0.877). However, a significant interaction between the posture and movement direction factors was observed (*F*(3, 78) = 13.406, *p* < 0.001, *η*_*p*_^2^ = 0.340), prompting post hoc analyses. In both the upright and lying conditions, rDPA was significantly shorter for upward than for downward movements (both *p* < 0.001). This result is consistent with findings of previous studies (Gaveau & Papaxanthis 2011; Gaveau et al. 2014) and suggests that participants exhibited similar behaviour in the virtual as in the normal physical environments. Additionally, in the upright condition, rDPA values were significantly greater for rightward than for leftward movements (*p* < 0.001). In contrast, rDPD did not meet the assumption of normality; therefore, a Friedman test was conducted, which revealed significance for this metric (*p* < 0.001). Post hoc Wilcoxon signed-rank tests showed that, in both virtual and physical conditions, rDPD was significantly shorter for upward than for downward movements in the lying posture conditions (for virtual environment, *p* = 0.002; for physical environment, *p* < 0.001).

For MD, normality tests indicated that the assumption of normality was not met across conditions. Consequently, a Friedman test was conducted, which revealed significance in the data (*p* < 0.001). However, subsequent Wilcoxon signed-rank tests showed no significant differences between any pair of conditions (comparison no.1–16, all *p* > 0.007 [corrected α threshold = 0.003125]).

For PV, the normality assumption was satisfied, and thus three-way repeated-measures ANOVA was performed. The results did not indicate any significant interactions across three factors (*p* = 0.565), between the visual environment and posture factors (*p* = 0.510), and between the visual environment and movement direction factors (*p* = 0.238). However, a significant interaction between posture and movement direction factors was observed (*F*(3, 78) = 33.887, *p* < 0.001, *η*_*p*_^2^ = 0.566). Post hoc analyses revealed that, in the upright conditions, PV was significantly greater for leftward than for rightward movements (*p* < 0.001). In the lying conditions, PV was greater for rightward than for leftward movements (*p* < 0.001), and for downward compared to upward movements (*p* = 0.0124).

Since both PA and PD met the assumption of normality, three-way repeated-measures ANOVAs were conducted for each. We observed no significant interactions across three factors (for PA, *p* = 0.555; for PD, *p* = 0.899), between the visual environment and posture factors (for PA, *p* = 0.965; for PD, *p* = 0.947), and between the visual environment and movement direction factors (for PA, *p* = 0.451; for PD, *p* = 0.587). However, significant interactions between posture and movement direction were observed for both parameters (for PA, *F*(3, 78) = 23.844, *p* < 0.001, *η*_*p*_^2^ = 0.478; for PD, *F*(2.079, 54.053) = 5.183, *p* = 0.008, *η*_*p*_^2^ = 0.166). For PD, the Greenhouse–Geisser correction was applied due to a violation of the sphericity assumption. Post hoc analyses showed that, for PA, values were greater for leftward than for rightward movements in the upright condition (*p* < 0.001), whereas in the lying condition, values were greater for rightward than for leftward movements (*p* < 0.001). For PD, in the lying condition, values were greater for downward compared to upward movements (*p* = 0.001), and for rightward compared to leftward movements (*p* < 0.001).

**Fig. 6:**
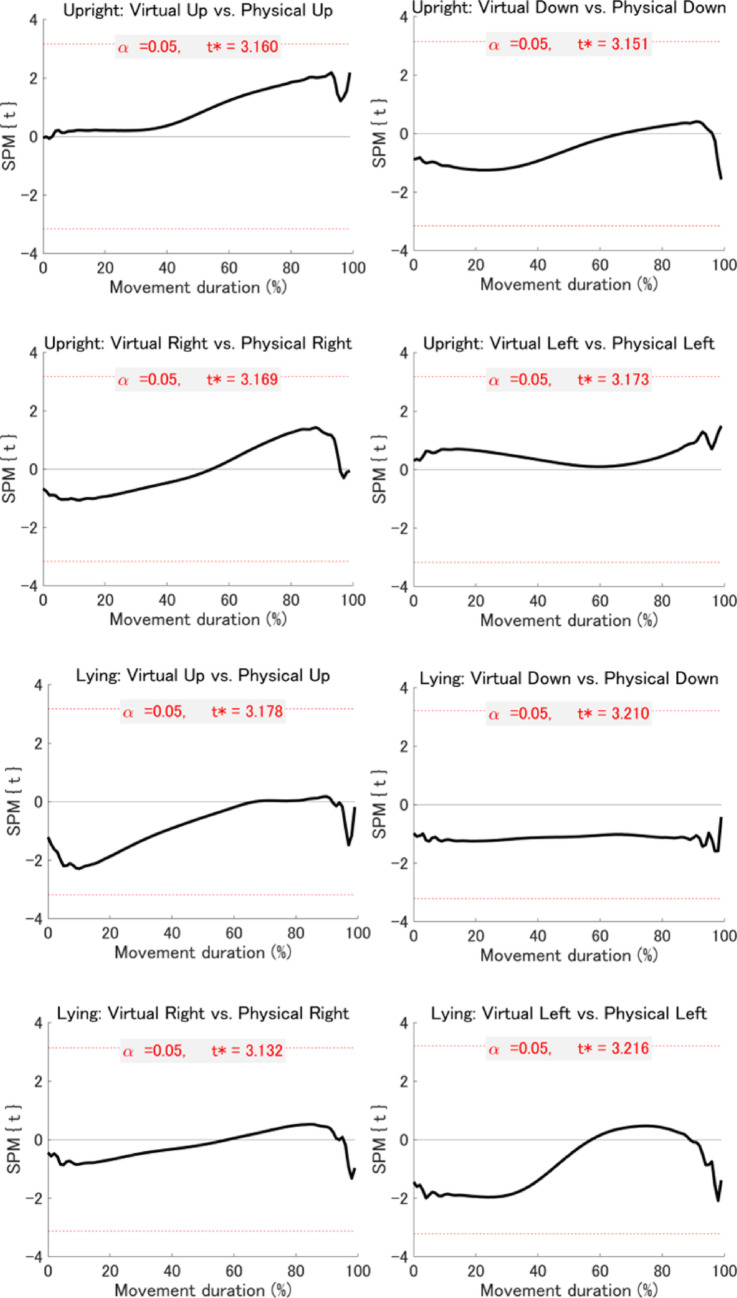
Results of the SPM analysis comparing velocity profiles between virtual and physical environments across posture and movement direction conditions. Each subplot shows the SPM{t} statistic (a t-value calculated at each time point) for a specific movement direction under a given posture (black line), with the significance threshold (t*) at α = 0.05 indicated by dashed red lines. The areas where the black line crosses the dotted red line (gray area) indicate suprathreshold clusters—contiguous time segments where the SPM{t} statistic exceeds the threshold, indicating statistically significant differences between conditions

**Fig. 7:**
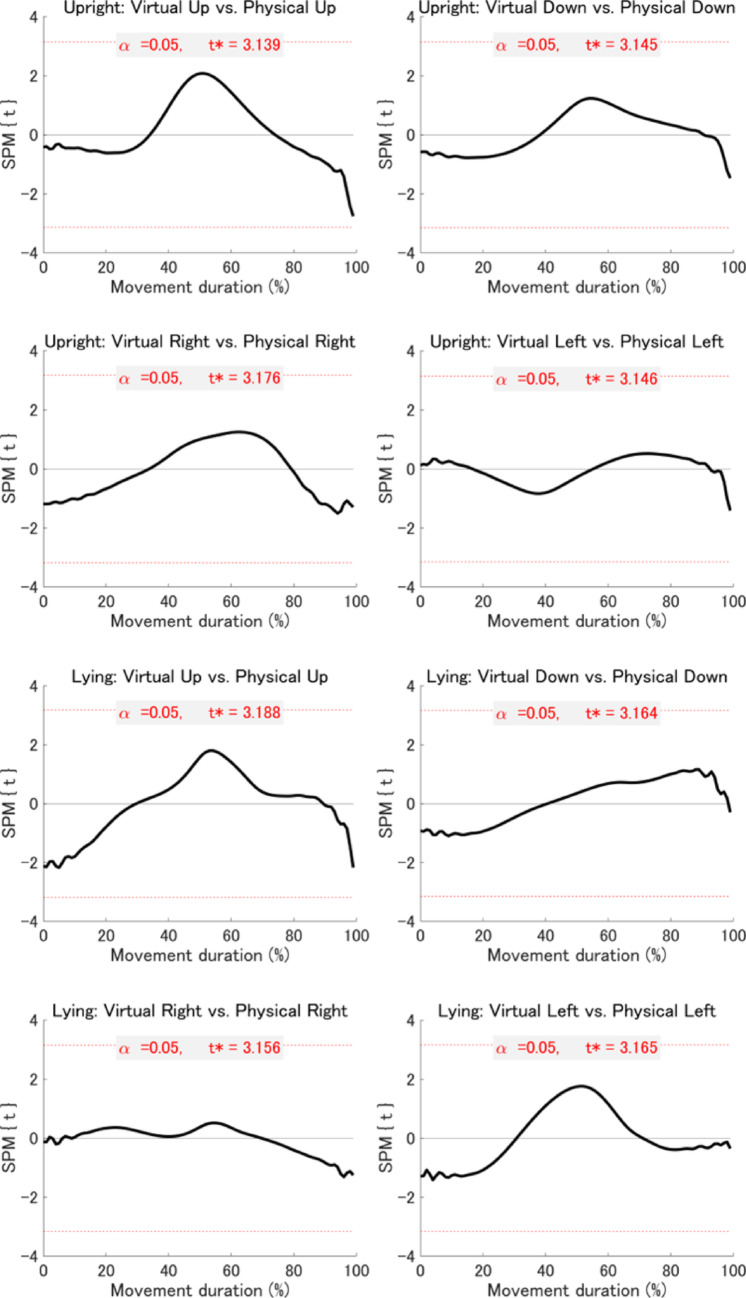
Results of the SPM analysis comparing acceleration profiles between virtual and physical environments across posture and movement direction conditions. Each subplot shows the SPM{t} statistic (a t-value calculated at each time point) for a specific movement direction under a given posture (black line), with the significance threshold (t*) at α = 0.05 indicated by dashed red lines. The areas where the black line crosses the dotted red line (gray area) indicate suprathreshold clusters—contiguous time segments where the SPM{t} statistic exceeds the threshold, indicating statistically significant differences between conditions

**Fig. 8:**
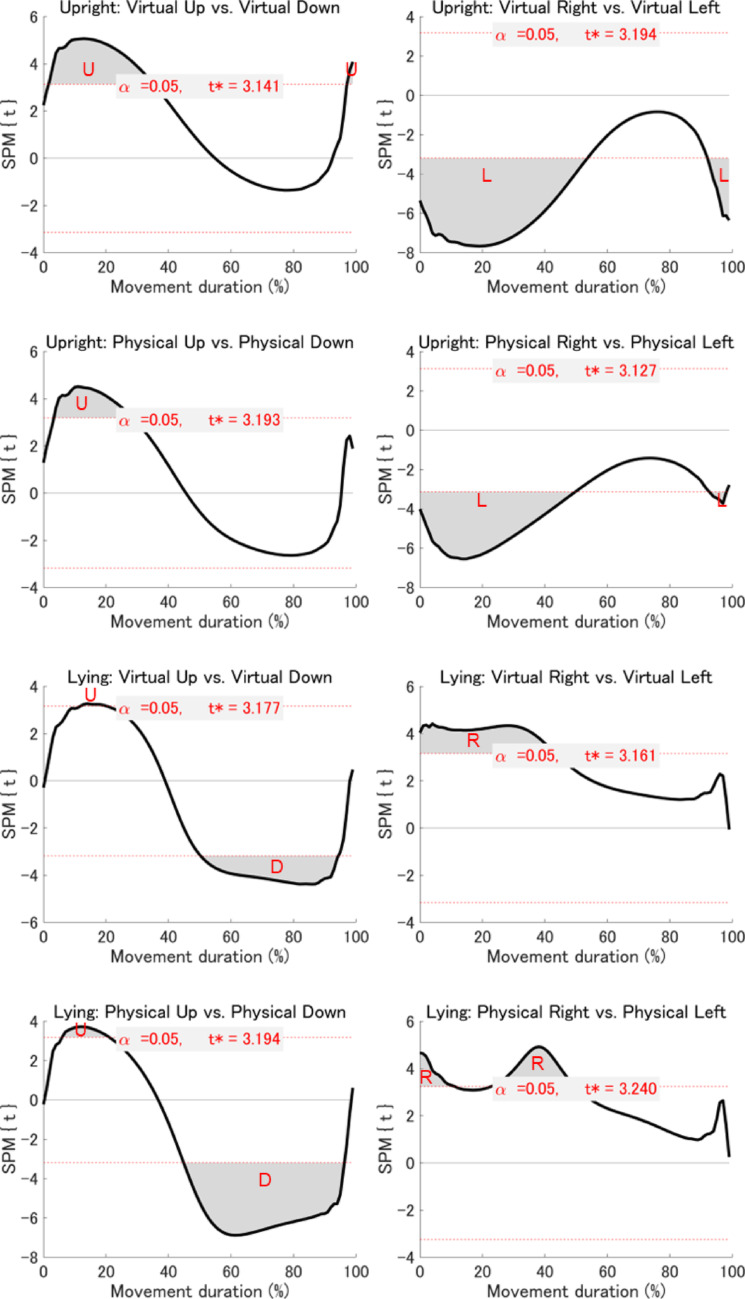
Results of the SPM analysis comparing velocity profiles between movement directions across visual environment and posture conditions. Each subplot shows the SPM{t} statistic (a t-value calculated at each time point) for a specific movement direction under a given posture (black line), with the significance threshold (t*) at α = 0.05 indicated by dashed red lines. The areas where the black line crosses the dotted red line (gray area) indicate suprathreshold clusters—contiguous time segments where the SPM{t} statistic exceeds the threshold, indicating statistically significant differences between conditions. Significant differences: U = upward > downward; D = upward < downward; R = rightward > leftward; L = rightward < leftward

**Fig. 9:**
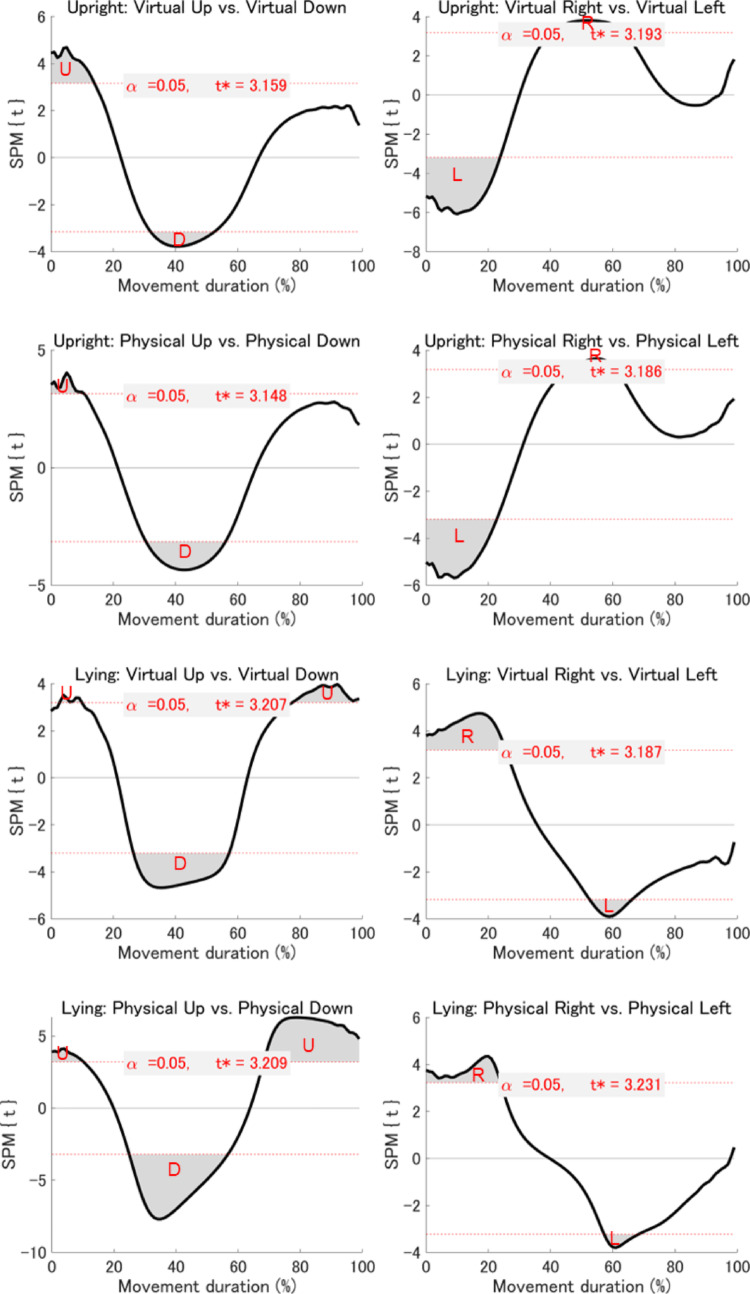
Results of the SPM analysis comparing acceleration profiles between movement directions across visual environment and posture conditions. Each subplot shows the SPM{t} statistic (a t-value calculated at each time point) for a specific movement direction under a given posture (black line), with the significance threshold (t*) at α = 0.05 indicated by dashed red lines. The areas where the black line crosses the dotted red line (gray area) indicate suprathreshold clusters—contiguous time segments where the SPM{t} statistic exceeds the threshold, indicating statistically significant differences between conditions. Significant differences: U = upward > downward; D = upward < downward; R = rightward > leftward; L = rightward < leftward

*Statistical parametric mapping (SPM) analyses*: To extend the summary kinematic measures reported above, we conducted an exploratory time-resolved analysis using SPM. While traditional measures focus on discrete time points (e.g., PA, PV, and PD), SPM enables continuous, fine-grained comparisons across the entire velocity and acceleration profiles. This approach allowed us to identify subtle, temporally specific differences between virtual and physical environments that may not be captured by conventional analyses. Accordingly, SPM analyses were performed on the velocity and acceleration profiles of the index finger for the 16 planned comparisons described above. The results revealed no significant differences in either velocity or acceleration profiles between virtual and physical environments at any temporal point during the movement (Figs. 6 and 7). However, when comparing upward with downward movements and rightward with leftward movements within each combination of visual environment and postural condition, significant differences were observed across all eight combinations for both velocity and acceleration profiles (Figs. 8 and 9). While we emphasise that our SPM analyses were exploratory in design, and must therefore be interpreted with relative caution, these findings highlight directional differences in kinematics that are not easily captured by the discrete kinematic landmarks described above.

## Discussion

The present study aimed to investigate whether motor control in a virtual environment differs from that in a physical environment, with a specific focus on gravity-related control of upper-limb pointing movements. To this end, participants performed arm-pointing tasks in four directions (upward, downward, rightward, and leftward, from an allocentric perspective) under both upright and lying postures, across virtual and physical environment conditions. Our results showed that rDPV—a well-established kinematic parameter of gravity utilisation—was consistently shorter for upward than for downward movements in *both* virtual and physical environments, while no significant differences were observed between rightward and leftward directions. These findings replicate previous results (Gaveau et al. 2014, 2021; Gentili et al. 2007; Papaxanthis et al. 2005; Sciutti et al. 2012; Yamamoto and Kushiro 2014), support our hypotheses (H2 and H3), and confirm an inherent effect of gravity on the planning and control of upper-limb movements in both visual environments. However, contrary to our hypothesis (H1), which predicted a difference in rDPV between virtual and physical environments for both upward/downward and leftward/rightward movements, no significant differences were observed between the two visual environments when posture and movement direction were held constant. Similarly, exploratory SPM analyses of velocity profile revealed no significant differences between virtual and physical environment conditions across the movement period. These consistent patterns of data are striking, given the remarkably coherent kinematic profiles displayed between the two environments, and the fact that participants spontaneously behaved in this manner (i.e., they were not told to point in any particular way in the virtual environment and there was no actual requirement for gravity to exist in this virtual world). Therefore, these results indicate that gravitational effects on pointing movements can be reliably evaluated in virtual environments, just as in physical environments.

Consistent with the findings for rDPV, and the results of our exploratory SPM analyses, no statistically significant differences between virtual and physical environments were observed for any of the other kinematic parameters. Which raised the question as to why previous studies have reported differences between virtual and physical environments. One possible explanation lies in the motor control strategy. In previous studies where slower movement was observed in virtual compared to physical environments, the movement tasks involved prehension movements (Arlati et al. 2022; Furmanek et al. 2019; Magdalon et al. 2011). In contrast, the current study employed a pointing task in which participants simply pointed toward a target. Compared to prehension movements, pointing movements are considered to rely more heavily on feedforward control (Carnahan et al. 1993). Indeed, previous studies examining vertical pointing movements that are similar to those in the present study, suggested that feedforward processes are more heavily involved in generating the direction-dependent kinematic differences in such movements (Gaveau and Papaxanthis 2011; Poirier et al. 2020). In addition, the instruction given to participants to perform arm movements as quickly and accurately as possible, without any corrections during execution, may also have contributed to a greater reliance on feedforward processes. Thus, participants in the current study likely relied more on ballistic, feedforward control rather than on feedback based on online visual and somatosensory inputs.

An alternative but related explanation is that the previous studies reporting slowed movements (Arlati et al. 2022; Furmanek et al. 2019; Magdalon et al. 2011) involved impoverished endpoint haptic feedback. For example, grasping with a virtual reality controller (Arlati et al. 2022), using haptic gloves (Magdalon et al. 2011), or a total absence of endpoint feedback (Furmanek et al. 2019). In contrast, our task required no terminal contact, ensuring that the sensory conditions in the virtual and physical environments were closely matched, which may have further contributed to the congruence of physical and virtual behaviours. Combined with the reliance on feedforward control in the vertical pointing task and the knowledge that gravity still exists in their surroundings (irrespective of whether they are present in the virtual environment or not), this may explain why no significant kinematic differences were observed between virtual and physical environments.

As for rDPA, a significant interaction between posture and movement direction factors was observed. Subsequent post hoc tests revealed that rDPA was significantly shorter for upward than for downward movements in both the upright and lying posture conditions. This result replicates findings from previous studies (Gaveau and Papaxanthis 2011; Poirier et al. 2022) and suggests that direction-dependent kinematic asymmetries emerge at an earlier phase of movement than rDPV. Further supporting evidence was provided by the exploratory SPM analysis which showed that, in both virtual and physical environment conditions under the same posture and visual environment, velocity and acceleration were greater for upward than for downward movements during the very early phase of the movement (i.e., before peak acceleration), which could not be captured using summary kinematics. SPM analysis enables a more detailed examination of movement profiles than summary measures and should therefore be employed in future studies. In any case, these early-phase kinematic asymmetries suggest that greater feedforward motor output is required to move the upper limb upward against gravity.

One limitation of the present study lies in the distance from start position to target positions. In this study, the field of view by the head-mounted display (HMD) used in the virtual environment condition may have restricted the visibility of the targets. To ensure that the targets remained within the HMD’s field of view, the distance between the start position and the target was fixed at 30 cm, but asymmetries in the specific field of view for each participant was difficult to quantify, particularly when laying down. Therefore, it would be valuable for future research to investigate how kinematic parameters that reflect gravity-related motor control strategies may change when performing movements over longer distances. Additionally, this study did not include measurements of muscle activity, and thus cannot provide direct physiological evidence regarding the underlying mechanisms. Previous studies measuring muscle activity during vertical pointing movements in the physical environment demonstrated that participants utilised the gravity to reduce muscle effort (Gaveau et al. 2021; Poirier et al. 2022). To determine whether physiological mechanisms differ between virtual and physical environments, future research should incorporate a variety of kinematic and muscle activity measurements.

Another factor to consider is the temporal delay associated with virtual reality systems. Head-mounted displays can introduce small latencies between participant movements and visual feedback, typically on the order of 20–40 ms for modern devices under optimal conditions (including motion-to-photon latency) (Warburton et al. 2023). While predictive algorithms help reduce the perceptual impact of these delays, factors such as rapid hand or head movements, controller tracking, or reductions in display refresh rates can introduce additional minor variability which could influence physical versus virtual comparisons. However, the highly similar kinematic profiles between virtual and physical conditions observed here suggest that any system delays were negligible in the context of feedforward-dominated pointing tasks which are relatively insensitive to modest visual delays (Desmurget and Grafton 2000).

## Conclusion

This study investigated whether upper-limb pointing movements that account for gravitational effects differ between virtual and physical environments. By analysing various kinematic parameters and applying exploratory SPM analysis, we found no differences between virtual and physical environments in the execution of pointing movements across different postures and movement directions. Importantly, in both visual environments, rDPV and rDPA were consistently smaller for upward compared to downward movements—replicating previous findings and suggesting that motor control strategies that account for the effects of gravity are preserved in virtual environments in the same way as physical environments, supporting the use of virtual and augmented reality as valid tools for studying motor control of pointing under controlled experimental conditions. Future research should examine longer movement distances and include physiological measurements to further explore the underlying mechanisms of an inherent effect of gravity on the planning and control of pointing movements.

## Data Availability

All relevant data and code is available online from: https://osf.io/rgvuk/

## Footnotes

1 Even without applying the Bonferroni correction, no pairs were found to have a *p*-value below 0.05. Only the comparison between virtual and physical environments for upward movements in the upright posture showed a *p*-value of 0.093, while all other comparisons between virtual and physical environments under different conditions yielded *p*-values greater than 0.248.
